# Yak Gut Microbiota: A Systematic Review and Meta-Analysis

**DOI:** 10.3389/fvets.2022.889594

**Published:** 2022-06-28

**Authors:** Yuxin Su, Junhong Su, Fanglin Li, Xiaojing Tian, Zewen Liu, Gongtao Ding, Jialin Bai, Zhuo Li, Zhongren Ma, Maikel P. Peppelenbosch

**Affiliations:** ^1^China-Malaysia National Joint Laboratory, Biomedical Research Center of Northwest Minzu University, Lanzhou, China; ^2^Department of Gastroenterology and Hepatology, Erasmus MC – University Medical Center, Rotterdam, Netherlands; ^3^Ganan Research Institute of Yak Milk, Hezuo, China

**Keywords:** yak, environment factors, gut microbiota, rumen microbiota, yak husbandry

## Abstract

The yak (*Bos grunniens*) is closely related to common cows (*Bos taurus*), but is clearly a distinct species. Yaks are of substantial importance to food and leather production in certain high-altitude regions of Asia. The animal is increasing elsewhere as well, mainly because of the perceived health benefits of its milk. Like all ruminants, the animal harbors a complex community of microbial cells in its gut, crucial for its physiology. Despite yaks being important domestic animals, the composition of its gut microbiota and how the composition is guided by its specific high-altitude environment remains largely uncategorized. Hence, online databases (Embase, Medline ALL, Web of Science Core Collection, Cochrane Central Register of Controlled Trials, and Google Scholar) were searched for articles on yak intestinal microbiota. The pooled taxonomic abundance was compared between regions, sexes, different age groups, and feeding patterns. The gut microbiota distribution across different yak intestinal segments was established through pooled average taxonomic abundance. A total of 34 studies met the inclusion criteria and yielded information on 982 unique yak gut microbiota samples. An analysis of overall pooled microbiota revealed a segmented microbial community composition of the yak gut. Yak rumen microbiota was significantly influenced by difference in region, sex, and feeding patterns, the latter factor being dominant in this respect. Yak microbiome is shaped by the feeding strategy and provides an obvious avenue for improving health and productivity of the animal. More generally, the current segmental description of physiological gut microbiome provides insight into how the microbiology of this animal has adapted itself to help comping yaks with its high-altitude habitat.

## Introduction

The gut microbiome constitutes a complex and vital ecosystem in all mammals, but being responsive for nutrient extraction and production following the consumption of cellulose enriched Poaceae, especially so for cattle species (1, 2). Much is known on the microbiota composition of cows, but for other species of bovine family, such information is largely lacking. Nevertheless, knowledge on the microbiota of non-cow bovine family members may provide important insights as to how specific environments drive microbiota composition and provide important clues as to how to improve the health and economic production from such animals. The paucity of knowledge on non-cow bovine family members also holds true for yaks, a unique ruminant animal mainly found in the Qinghai-Tibet Plateau. As it lives at altitudes ranging from 3,000 to 5,500 m, the animal is well adapted to hypoxia, lower temperatures, and specific feedstuffs (3, 4). A number of recent studies have provided evidence that yak gut microbiome may play a key role in their adaptation to this harsh environment even as the composition of the yak microbiome remains poorly categorized (5). Understanding yak microbiota gains further importance by the increased herding of yaks in other continents, also driven by the premium payment for yak milk. Thus, a variety of considerations prompts further understanding of the yak microbiota composition.

Like all bovine family members, the yak intestine is dominated, both physically and physiologically, by its rumen. The yak rumen is characterized relative to other than other intestinal segments by its high bacterial load of microbiota. The yak rumen is the largest compartment among all the intestinal segments and the microbiota of this body part enables the animal to digest plant fibers and non-fiber carbohydrate and represents the major site of fermentation in yaks. As the nexus of its physiology, understanding the factors that affect microbiota composition of the yak rumen are essential for devising rational avenues for improving health and well-being of this animal and enhancing economic value. A systematic study on these factors is thus necessary to advance the field.

Here we conducted a systematic review and meta-analysis to extensively evaluate the effects of the regional distribution, feeding pattern, sex, and age groups on yak rumen microbiota. We show that the taxonomical composition of yak rumen microbiota was significantly affected by these external and internal factors, and, to our knowledge, this study is the first work that provides a full description of microbiota distribution in the entire intestinal system of yak.

## Methods

### Search Strategy

International online databases (Embase, Medline, Cochrane, Web of Science, and Google Scholar) were used to search articles on yak microbiota in the English language from inception until June 2020. The terms used in search of different databases were ('yak'/de OR (yak OR yaks OR bos-grunnien^*^):ab,ti) AND ('microflora'/exp OR (microflora OR microbiota OR flora OR microbiom^*^):ab,ti) for Embase, ((yak OR yaks OR bos-grunnien^*^).ab,ti.) AND (exp Microbiota/ OR (microflora OR microbiota OR flora OR microbiom^*^).ab,ti.) for Medline ALL, TS=(((yak OR yaks OR bos-grunnien^*^)) AND ((microflora OR microbiota OR flora OR microbiom^*^))) for Web of Science Core Collection, ((yak OR yaks OR bos-grunnien^*^):ab,ti) AND ((microflora OR microbiota OR flora OR microbiom^*^):ab,ti) for Cochrane Central Register of Controlled Trials, and yak|yaks| “bos^*^grunnien” microflora|microbiota|flora|microbiome for Google Scholar.

### Inclusion and Exclusion Criteria

As proposed previously by others (6, 7), for inclusion, selected scientific literature had to report quantitative or relative quantitative information on the abundance of microbiota taxa in the yak paunch or other parts of its stomach and/or of other intestinal compartments in this animal. The information reported ranges from the phylum to the genus level. Only English-language research articles were included, while articles without full text and abstract, duplicated studies, conferences, review articles, and editorial reports were excluded.

### Data Extraction

After collecting findings from all databases, the articles were exported to a reference manager (EndNoteX7; Thomson Reuters). Duplicates were removed automatically by the software and by hand. Two reviewers (YX and FL) independently screened the titles and abstracts of all included articles to determine their eligibility. Any disagreement was handled by the third reviewer (JH) and consensus was reached through discussion between all three reviewers. The microbiota data were extracted from the included studies and recorded in the form of relative taxonomical abundance for further analysis. Moreover, a specific software (GetData Graph Digitizer, v 2.25) was used to extract the raw data from graphs if no visual data were available. The range of data extraction was set up to include only the phylum-level and genus-level relative abundances of yak gut microbiota. For a qualified analysis and comparison, only healthy yaks were included for data extraction, while unhealthy yaks (e.g., diarrheal and growth retarded) or yaks treated with specific diet regime (e.g., starvation and fattening) were excluded. Second, to ensure a robust downstream analysis, the resources of all samples that included in this study had to be consistent for each segment of yak digestive system. The information on environmental factors involved in this study was provided in detail in Supplementary Table S1.

### Data Analysis

Following the data collection, further processing was performed in Microsoft Excel v.2016 MSO. The information extracted from the included studies was converted into relative abundance expressed as percentages of bacteria observed in stomach (reticulum, rumen, omasum, and adomasum), duodenum, jejunum, ileum, cecum, colon, and feces of the yak and finally yielded mean data and CIs on microbiota composition in different locations of the yak gut. Following conversion of all extracted data to a standardized reporting format, the relative abundance and SD, the overall relative abundance of bacteria from different phyla to genus was established. For each bacterial taxon, the difference in their relative abundance between different groups was calculated. The Mann–Whitney test was used for the two-group comparison, while multiple comparisons and *post hoc* test were performed using the Kruskal–Wallis test and Dunn test, respectively. A *p*-value was considered significant if it is <0.05. Finally, the yak gut microbiota variation explained by environmental factors that were taken into consideration in this study was assessed using the function envfit in the vegan package of R v4.2.1, and the significance of the fit was calculated using a permutation approach (perm = 999).

## Results

### Study Selection and Microbiota Data Extraction

**Figure 1:**
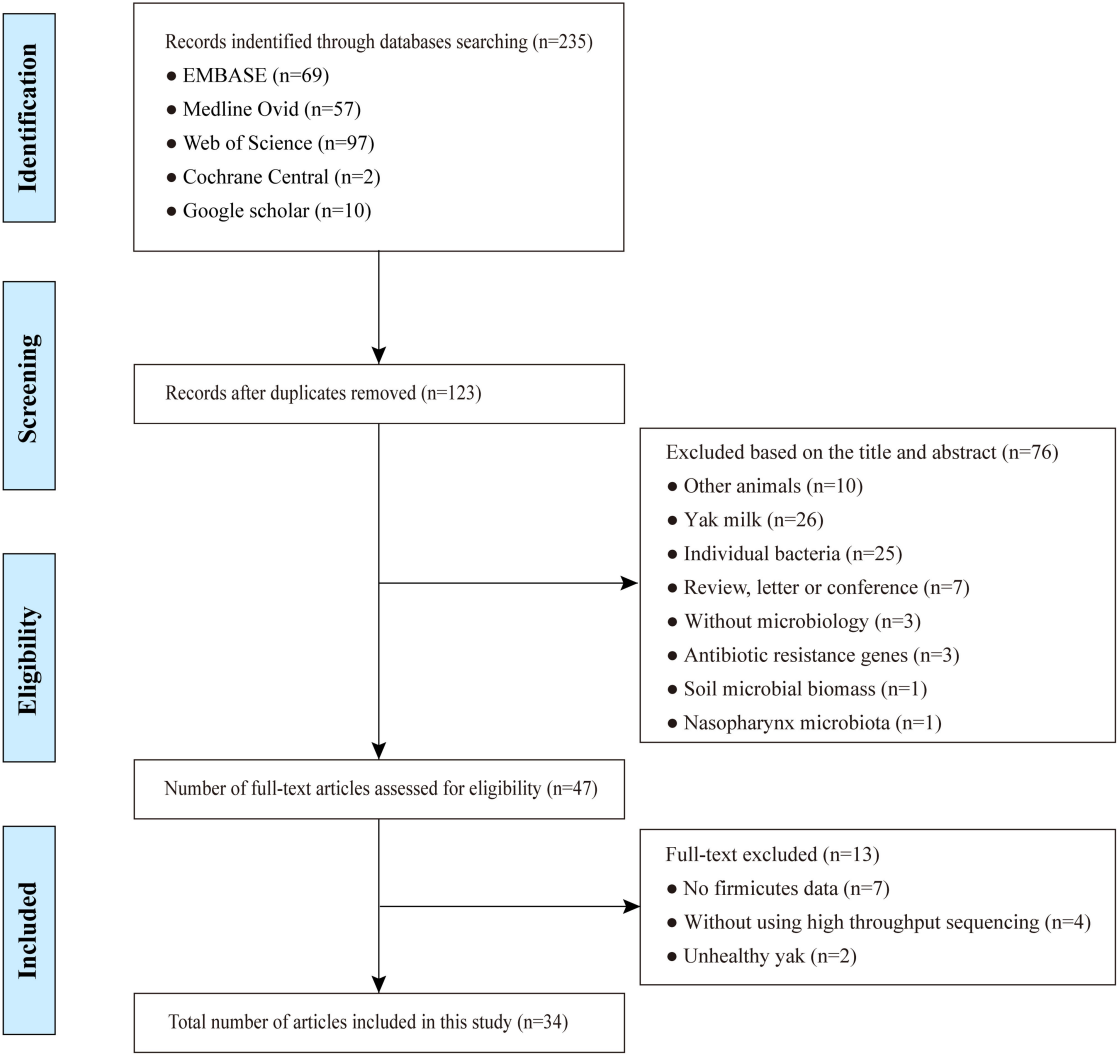
Flowchart diagram of screening and selection processes.

Following the initial internet search, a total of 235 studies were retrieved by querying Embase, Medline, Cochrane, Web of Science, and Google Scholar with our search criteria on yak gut microbiota (Supplementary Table S2). Following elimination of duplicates, 123 articles remained. Out of the remaining studies, 76 records were excluded after review of their titles and abstracts. The resulting 47 full-text studies selected for the initial analysis were further evaluated for compliance to the pre-defined inclusion criteria, which led to a further exclusion of 13 studies that did not meet these criteria. In the end, 34 studies including a total of 982 samples (Supplementary Table S3) that met the inclusion criteria underwent the final systematic review and meta-analysis (Figure 1). During data extraction, if no microbiota data were provided for individuals in a study, its mean value of a group of yak was used instead, but was considered one sample for this study. This resulted in a total of 294 samples finally included (Supplementary Table S3).

At the phylum level, more than three taxa were identified to be present in the yak gastrointestinal system, with three of them being reported for their relative abundance in all pooled sample results generated through our analysis, *in casu* the phyla Firmicutes, Bacteroidetes, and Proteobacteria. At the genus level (Supplementary Table S4), a total of eight genera identified as frequently reported genera in the yak gastrointestinal system were the genus *Succiniclasticum* [which can convert succinic acid into propionate and thus provide energy for the host (8)], *Prevotella* [which can degrade protein and hemicellulose to produce acetate and propionate (8)], and *Fibrobacter* [one of the major bacterial degraders of lignocellulosic material (9)]. Frequently reported in yak fecal samples are *Prevotellaceae_UCG-001, Prevotellaceae_UCG-003, Christensenellaceae_R-7, Rikenellaceae_RC9, Ruminococcaceae_NK4A214, Christensenellaceae_R-7, Rikenellaceae_RC9*, and *Ruminococcaceae_UCG-005*, which all are involved in fiber degradation. In addition, *Bacteroides, Akkermansia* [associated with improving the host metabolic functions and immune responses (10–12)], and *Ruminococcaceae_UCG-010* were detected as well. For the subsequent downstream analysis of this study, we selected these taxa as they appear as major representatives of the yak gut microbiome.

### Distribution of Yak Microbiota Across Different Gastrointestinal Segments

**Figure 2:**
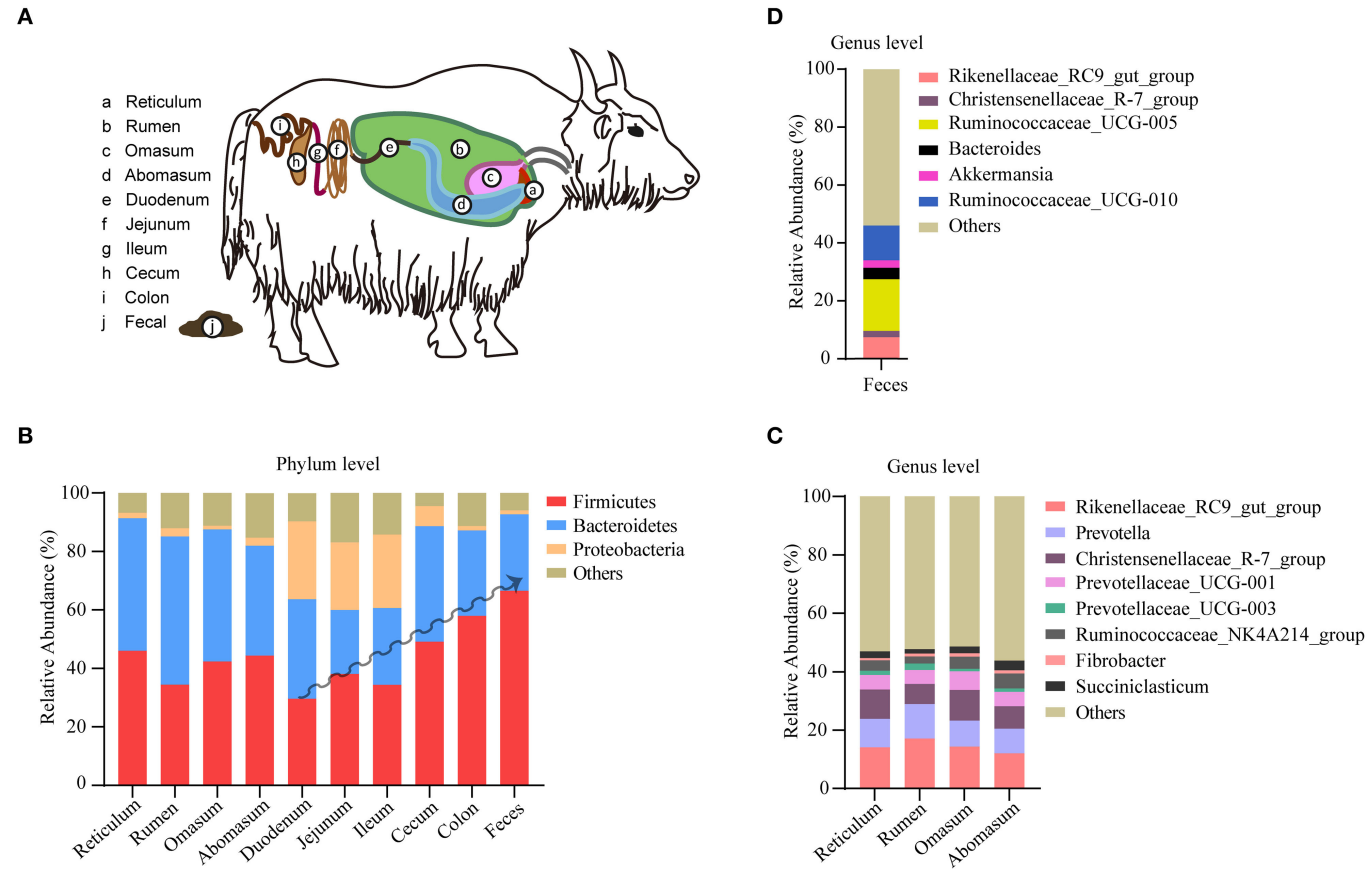
Microbiota distribution in different segments of yak digestive tract. **(A)** Overview of the segmented yak gastrointestinal system. **(B)** The composition of microbiota at phylum level in reticulum (*n* = 9), rumen (*n* = 481), omasum (*n* = 9), abomasum (*n* = 9), duodenum (*n* = 9), jejunum (*n* = 9), ileum (*n* = 9), cecum (*n* = 9), colon (*n* = 21), and feces (*n* = 417). **(C)** The composition of microbiota at genus level in rumen (*n* = 129), omasum (*n* = 9), reticulum (*n* = 9), and abomasum (*n* = 9). **(D)** The composition of microbiota at genus level in yak feces (*n* = 98). The relative abundance of each taxa was expressed as mean.

The yak's digestive tract can be described as a three-compartment system, consisting of a stomach compartment (rumen, reticulum, omasum, abomasum), a small intestinal compartment (duodenum, jejunum, ileum), and large intestine (cecum, colon, rectum; Figure 2A). In the stomach compartment, the rumen is the dominant substructure in which microbes ferment feed and produce volatile fatty acids and thus providing the yak's main source of energy. In this study, a total of 565 samples from different gastrointestinal segments were included and pooled for generating a comprehensive description of microbiota distribution across the major gastrointestinal segments in yak. A number of 417 samples from yak feces were also included for a comprehensive analysis of yak fecal microbiota. We observed a distinctive composition of microbiota between different yak gastrointestinal segments and feces (Figures 2B–D), in agreement with the situation observed in other members of the bovine family (13, 14). In the microbiota of the yak colon, the most dominant phylum with respect to its relative abundance is Firmicutes (59.05%), followed by Bacteriodetes (28.09%) and proteobacteria (1.26%). In particular, the abundance of Firmicutes shows an increasing trend toward the duodenum, the ileum, the cecum to the colon; although various, each compartment of the stomach shows similar level of abundance for these phyla (Figure 2B). By contrast, the Firmicutes phylum achieves the highest level in yak feces (Figure 2B). The largest stomach compartment of the yak, the rumen, dominates the physiology of animal and its microbiota was characterized by a high abundance of Bacteriodetes (52.25%) and Firmicutes (31.24%). At the genus level, the genera *Rikenellaceae_RC9, Prevotella, Christensenellaceae_R-7*, and *Prevotellaceae_UCG-001* were the most dominant in the four stomach compartment (Figure 2C). *Prevotella* was highly prevalent in the rumen, but its levels showed a decreasing trend toward the reticulum and abomasum (Figure 2C). In fecal samples, the most dominated genera were *Rikenellaceae_RC9, Ruminococcaceae_UCG-005*, and *Ruminococcaceae_UCG-010* (Figure 2D). Our findings show that different elements of the yak gastrointestinal tract are characterized by different specialized microbiological ecosystems which is broadly consistent with the situation observed with respect to gut microbiome in cows (15).

### Geography Affects Yak Gut Microbiota Composition

**Figure 3:**
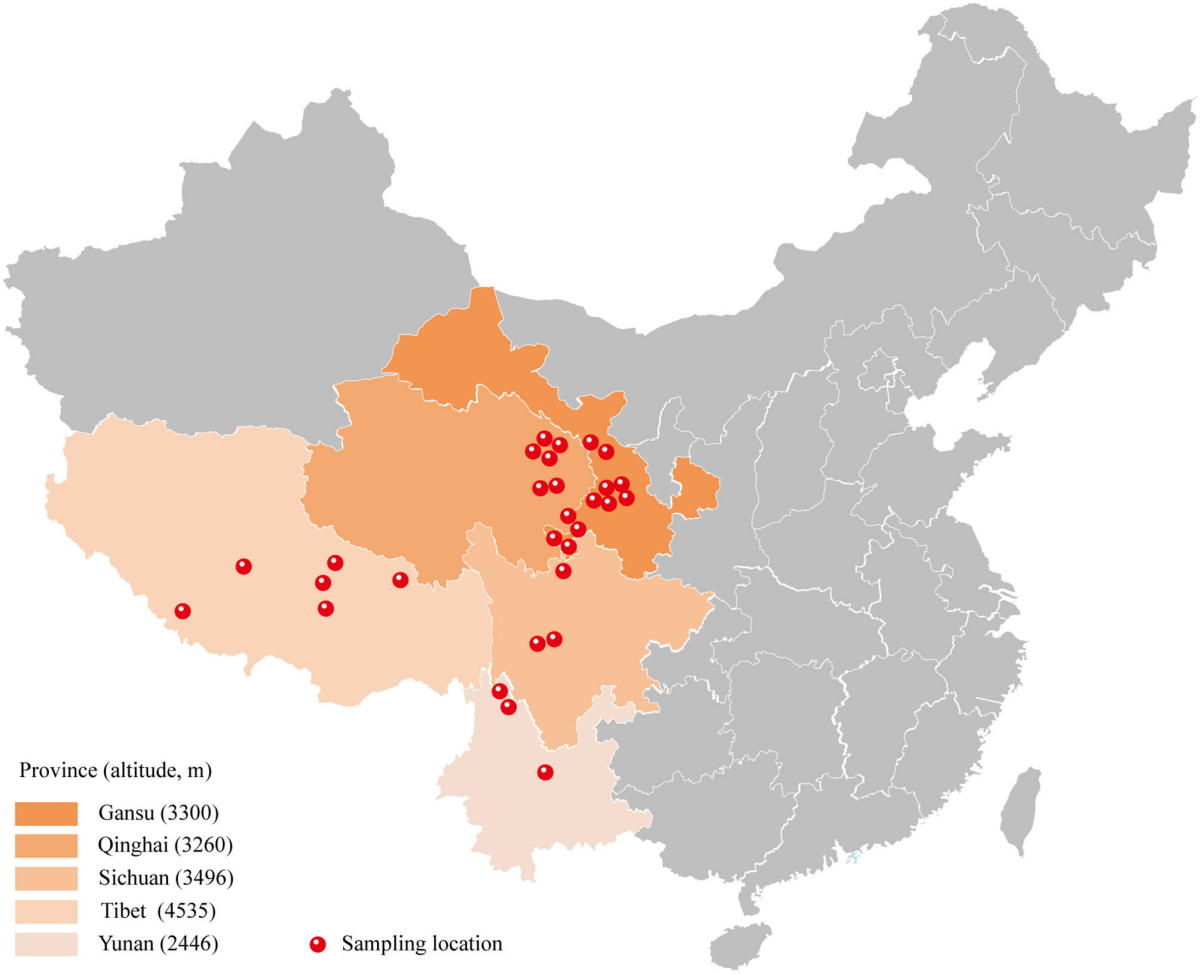
A map showing distinctive sampling locations in different areas. There are five provinces identified with different altitudes: Gansu province, Qinghai province, Sichuan province, Tibet, and Yunnan province.

**Figure 4:**
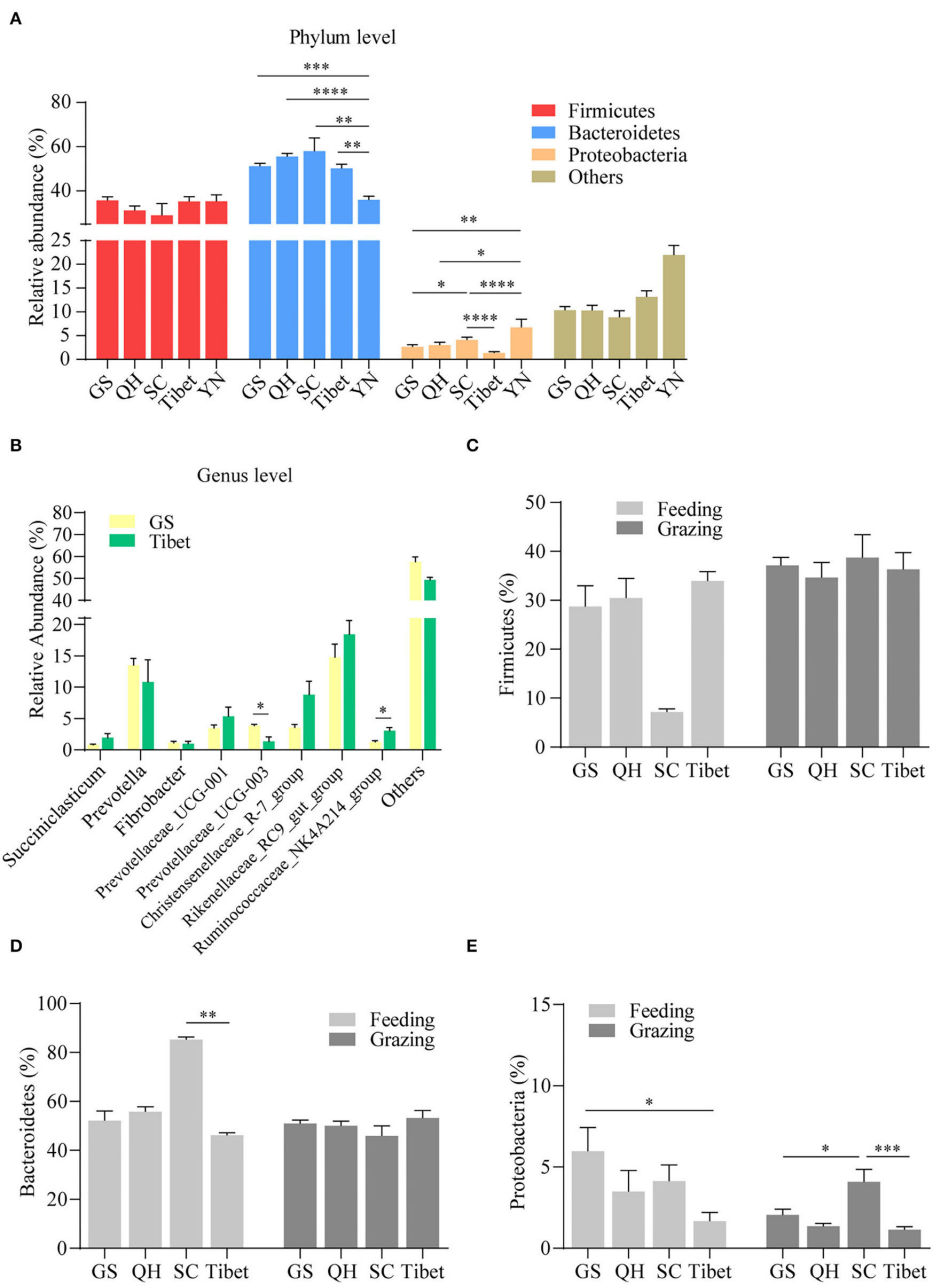
Regional influence on yak rumen microbiota. The relative abundance of phylum **(A)** and genus levels **(B)** in rumen microbiota in different provinces was compared, irrespective of the method of yak husbandry. Comparison of the relative abundance of the Firmicutes **(C)**, Bacteroidetes **(D)**, and Proteobacteria **(E)** in different provinces under the method of grazing vs. feeding. The data were expressed as mean ± SEM. The Mann–Whitney test was applied for intragroup comparison, whereas the Kruskal–Wallis test with *post hoc* Dunn's test was used for intergroup comparison. *p < 0.05, **p < 0.01, ***p < 0.001, ****p < 0.0001. GS, Gansu province; QH, Qinghai province; SC, Sichuan province; YN, Yunnan province.

Regional differences with respect to the properties of yak milk and physiology, in general, have been described, and hence, we were interested to see whether the topographical origin is reflected in the composition of the gut microbiota. Hence, the pooled microbiota data obtained on the yak rumen were stratified according to five distinct regions, *in casu* the Gansu Province, the Qinghai Province, the Sichuan Province, Tibet, and Yunnan province (Figure 3). The choice for the rumen microbiota for this analysis was mainly made because of practical considerations, as sufficient studies were available on this element of yak gastrointestinal tract to allow a meaningful analysis in this respect. Importantly, we observed a substantial influence of geographical location on the composition of the rumen microbiome, even on the phylum level, substantial difference being present in this respect (Figure 4A). When analyzed on the genus level, further distinctions became apparent, for instance between Gansu and Tibet (Figure 4B). Such regional differences may be explained by many different factors, but an important consideration is the alternative yak husbandry (16). Thus, we re-analyzed our results, also contrasting yak rumen microbiota obtained in grazing animals to those obtained from yaks maintained indoors. Interestingly, we found that on the higher taxonomic level, *in case* the Firmicutes (Figure 4C) and Bacteroidetes levels (Figure 4D), the composition of yak rumen microbiota no longer showed geographical dependence for grazing yaks, but on the taxonomically lower Proteobacteria level, differences in microbiota levels were still present between animals from the Gansu, Sichuan, and Tibet (Figure 4E). Strikingly, geographical differences in microbiota composition were enhanced, both on the Firmicutes and Bacteroidetes level, when only data for yaks kept indoors were contrasted (Figures 4C,D). Thus, although the mode of yak husbandry clearly influences the composition of gastrointestinal microbiota in this animal, other geographical factors drive alternative microbiome composition as well.

To investigate the regional influence on yak fecal microbiota, we analyzed the pooled microbiota data obtained from fecal samples to compare the results between different provinces. We observed a significant geographical difference in fecal microbiota composition at both phylum (Supplementary Figure S1A) and genus levels (Supplementary Figure S1B). Although it was not fully clear up to date how geography may change the fecal microbiota in yaks kept indoors, the results from yak kept grazing showed that the geographical influence on the composition of yak fecal microbiota was associated with the method of yak husbandry (Supplementary Figures S1C,D). These data indicate that the method of yak husbandry may play different roles in shaping yak rumen and fecal microbiota.

### Yak Rumen Microbiota in Different Age Groups

**Figure 5:**
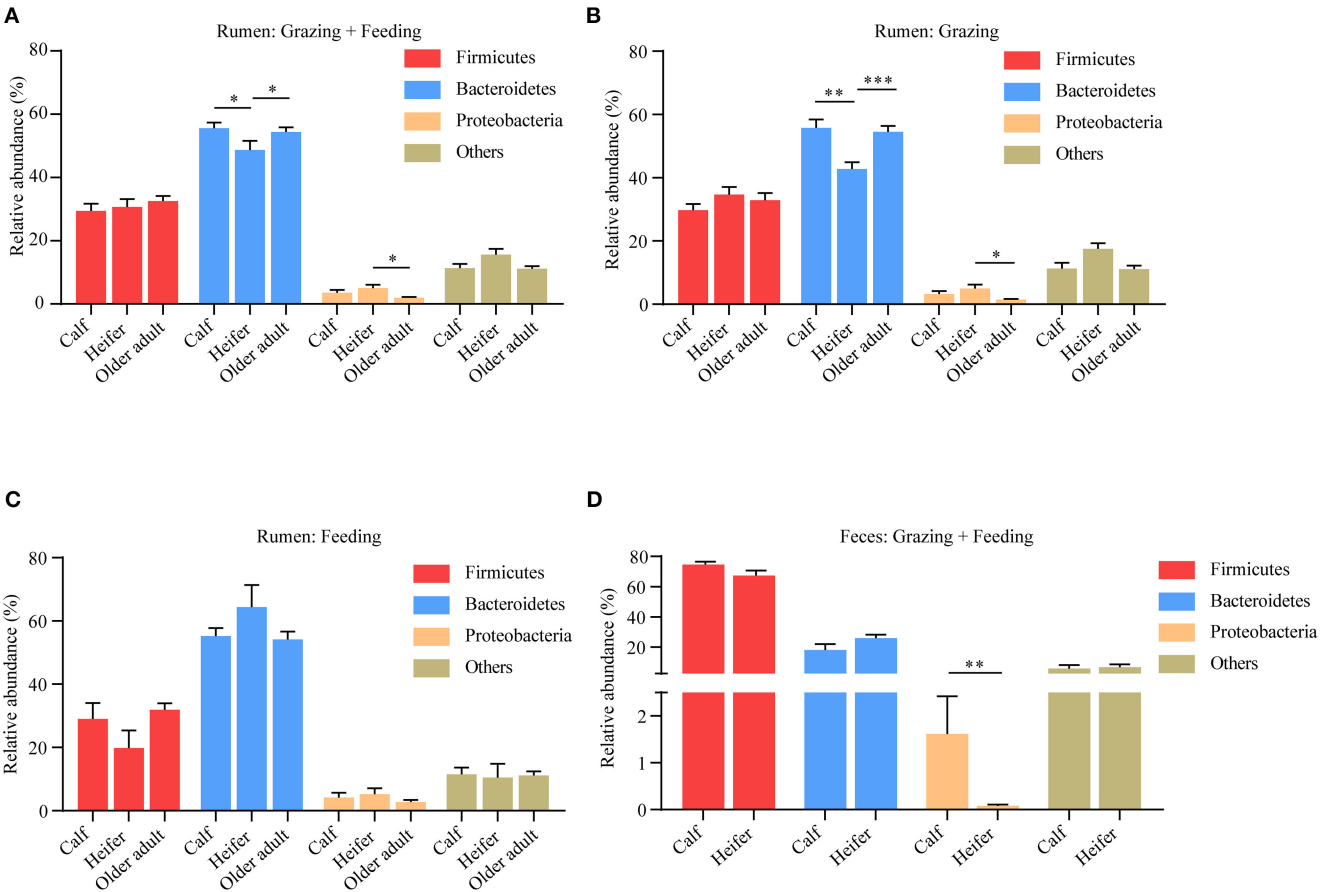
Influence of age on yak rumen and fecal microbiota. **(A)** The relative abundance at the phylum level of rumen microbiota was compared between age groups, irrespective of the method of yak husbandry. The difference of each phylum across different age groups when yak kept grazing **(B)** and indoor **(C)**. The data were expressed as mean ± SEM. The Kruskal–Wallis test with *post hoc* Dunn's test was used for multiple comparisons. *p < 0.05, **p < 0.01, ***p < 0.001. **(D)** Influence of age on yak fecal microbiota. The relative abundance at the phylum level was compared between age groups, irrespective of the method of yak husbandry. The data were expressed as mean ± SEM. The Mann–Whitney test was applied for availability of data. **p < 0.01.

For many species, the composition of microbiota shows a clear correlation with the age of the animal. To which extent this is also the case for yaks is not yet clear. Hence, we analyzed the effects of age on yak rumen microbiota. To this end, the pooled samples were divided into age groups: calves (aged <1 year), heifers (aged between 1 and 3 years), and older adults (aged more than 3 years). We found the abundance of Bacteroidetes was lower in heifers compared to calves and older adult yaks; however, the Proteobacteria was higher in heifers (Figure 5A). We also related our results to the mode of yak husbandry, contrasting rumen microbiota at different ages. Although again, at the phylum level, the composition of the rumen microbiota was different between the different age groups irrespective of the method of yak husbandry employed, now all differences between age groups emerged at the phylum level, especially between the heifer and the older adult group (Figures 5B,C). However, the abundance of Proteobacteria in fecal samples decreased in heifers without taking into account the method of yak husbandry (Figure 5D). These findings indicate that with regard to the composition of microbiota, age and mode of husbandry show substantial interaction and can only be understood when analyzed in conjunction.

### Rumen Microbiota of Yak of Different Sex

**Figure 6:**
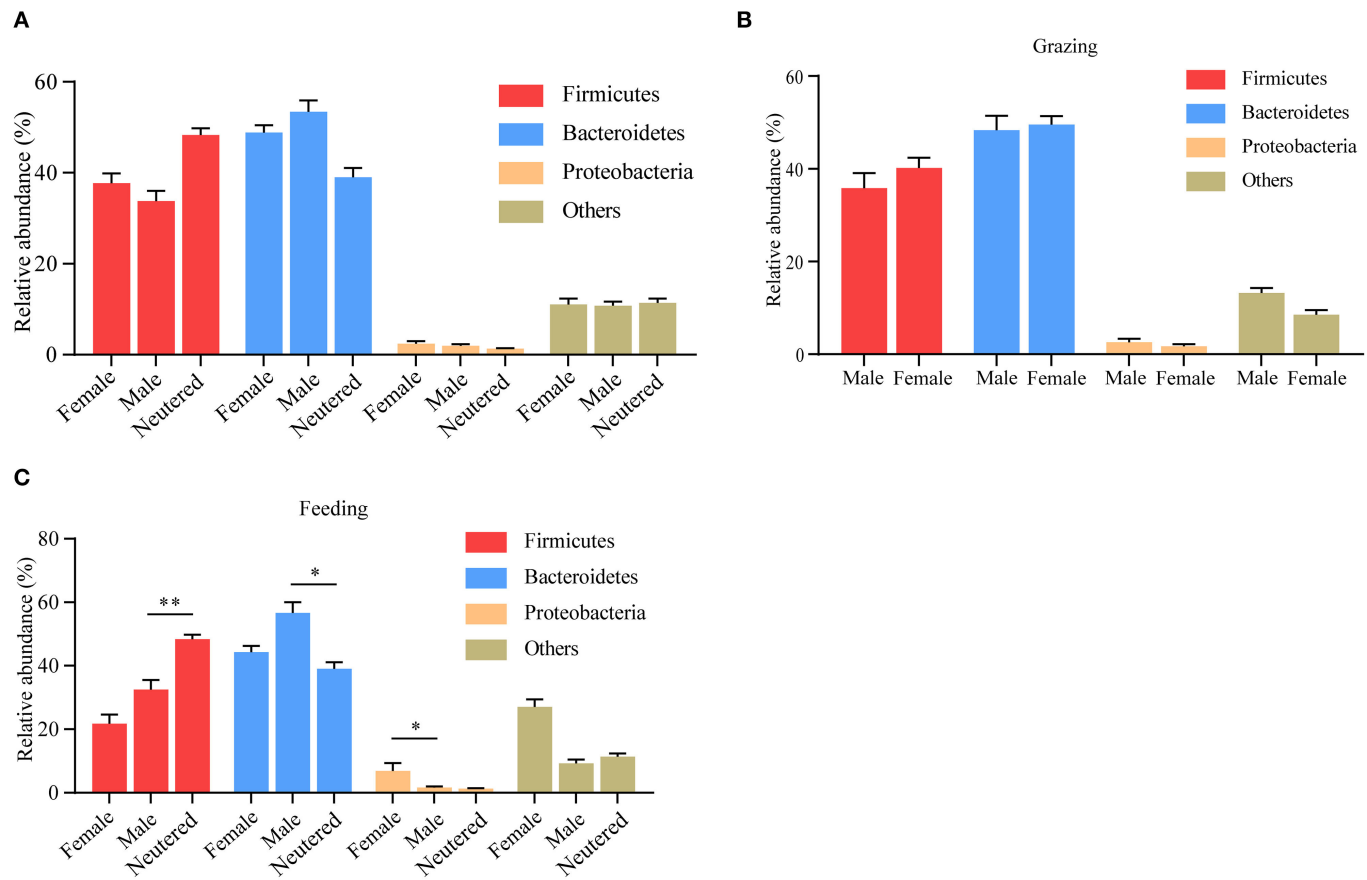
Influence of sex on yak rumen microbiota. **(A)** The relative abundance of phylum level was compared between male, female, and neutered yaks, irrespective of the method of yak husbandry. **(B)** The difference in each phylum when yak kept grazing. **(C)** The difference in each phylum when yak kept indoors. The data were expressed as mean ± SEM. The Mann–Whitney test was applied for intragroup comparison, whereas the Kruskal–Wallis test with *post hoc* Dunn's test was used for intergroup comparison. *p < 0.05, **p < 0.01.

Apart from diet and age, in many species, including humans, gut microbiota composition changes are also influenced by biological sex (17). The influence of sex, however, on the composition of yak microbiota remains largely obscure. Thus prompted, we analyzed the rumen microbiota data between male, female, and castrated animals. No differences in the composition of yak rumen microbiota were observed between male, female, and neutered animals (Figure 6A). To investigate whether the potential sex-dependent alternative microbiota composition was obscured by differences in yak husbandry pattern, we also performed this analysis following stratification in this respect. Although no difference was observed between sexes when yaks were kept outdoors (Figure 6B), differences become more pronounced between male and neutered animals that were maintained indoors (Figure 6C).

We further investigated the influence of sex on yak fecal microbiota. Unlike rumen microbiota, the influence of sex on yak fecal microbiota was pronounced, as indicated by an increase in Bacteroidetes in male yaks when paired with their female counterparts (Supplementary Figure S2A). However, this influence was lost when yaks kept grazing (Supplementary Figure S2B). At genus level, no difference was observed between male and female yaks irrespective of yak husbandry (Supplementary Figure S2C), although the relative abundance of *Bacteroides* was different between them when they kept grazing (Supplementary Figure S2D). Overall, our data show that the mode of yak husbandry is the major driver of yak gut microbiome composition and *per extenso* yak husbandry emerges a major factor driving physiology of this animal.

Lastly, gut microbiota variation explained by environmental factors was evaluated by employing the envifit function in R. We found the factors that appeared to be significantly associated with the Bray-Curtis distance-based composition of yak gut microbiota were batch effects (defined by the number of studies), intestinal segment, seasonal differences, sample location, sample location altitude, yak husbandry, feeding pattern, and age, while the contributions of province, sex, and physical condition were not significant (Supplementary Figure S3). Among the factors of which the contribution reached significance, the intestinal segment explained most of the variation of yak microbiota (29.5%). By contrast, seasonal difference only accounted for 4.47% (Supplementary Figure S3). Overall, the results of envifit analysis do not contradict the conclusions in the above.

## Discussion

Despite its regional importance and its growing economic importance, yak microbiota composition has been relatively poorly understood. Through the present comprehensive meta-analysis, we now provide an overview of the composition of this microbiota segmented to different elements of its gastrointestinal system, which all appear home to unique gastrointestinal ecosystems. In addition, we are able to analyze the various factors which may drive the microbiota composition in the yak rumen (which dominates the physiology of this animal). The mode of yak husbandry emerges as the main driver of the gut microbiome composition, although geographical differences, age, and sex hormonal status have influence as well. Although the results are influenced by trivial factors, such as batch effects, the envifit analysis generally supports these conclusions as well.

With respect to the influence of geographical differences on the microbiota composition, it is most straightforward to link these differences to altitude, both directly and indirectly through the alternative feed composition. For regions with different altitudes, the oxygen content of local atmosphere is varied and may have an important impact on the composition of yak gut microbiome. The presence in a hypoxic environment has been shown to affect the human intestinal microbiota (18), and it is thus not unreasonable to propose that a similar effect is present in yaks. In practice, pastures on which yaks roam often contain a fair amount of plants associated with specific altitudes, while the nutrient composition of plants may differ substantially at different altitudes, in turn, potentially affecting the rumen microbiota composition (19). Hence, we feel the effects seen that relate to geographical origin of the samples involved were not unexpected.

The effects observed associated with the sex status of the animal on the composition of yak rumen microbiota require further study as to its potential causes. In this study, we found that male yaks and neutered yaks display alternative composition of the rumen microbiota. Other studies, however, have provided evidence that sex is an important factor to influence the composition of gut microbiota in both humans and animals (20–22). Our data also appear to align well with the recent study of Barroso A et al. that shows that nutritional and hormonal disruption at early developmental periods perturbs the architecture of gut microbiota (23). Importantly, the contribution of sex to rumen microbiota change in yak was also affected by feeding patterns. Hence, it is possible that sex hormone–driven alterations of dietary preference of yaks drive the effects observed, but obviously further work is necessary to substantiate this notion.

A final observation that deserves further exploration is the age dependency of microbiome composition in yaks. Especially, the age-related changes in the relative abundance of Bacteroidetes in the rumen (which decreases from 58.66% in calves to 42.75% in heifers, and then increased to 57% in older adult yaks) are striking. These observations are in broad agreement with those reported in the recent by Zhaolong Nie et al. who observed that the proportion of Bacteroidetes in the rumen of juvenile Bazhou was lower than that in adult Bazhou (24). This observation is also in agreement with the dynamic changes of composition of the gut microbiota in cow with increased age (25). In addition, the genus *Prevotella* increases with age, which may reflect the switch from milk-based calorie intake to plant-based intake of calories, as this genus is associated with the breakdown of the associated fibers, although this notion obviously requires further validation. Overall, the cause of these effects remains unresolved, but may well relate to differences in feeding behavior.

Methane is a major component of greenhouse gas and directly contributes to global warming. As an indigenous animal, one of the important values of yak is its relationship with a low-methane environment. This is because yak yields lower level of methane than their low-altitude ruminants, such as cattle and sheep (26). Importantly, indoor feeding is a newly emerging factor that can elevate the methane-producing bacterial abundance in yak intestine system, as yak gut methane production showed an increased trend when they are exposed to indoor feeding patterns (27). Therefore, developing advanced feeding regimen to reduce methane-producing bacteria should receive more attention in this respect.

In conclusion, domesticated yaks, being a groups of animals of substantial local importance as a resource for milk, meat, and leather, and obtaining an increasing global importance in this respect, are characterized by a unique microbiome that shows substantial regional specification. External factors, in particular, not only the mode of husbandry but also geographical location, sex hormone status, and age, influence this composition. Knowledge on the factors that guide yak microbiota composition may yield novel rational avenues for designing strategies aimed at improving animal welfare and enhancing the economic value.

## Data Availability Statement

The data analyzed in this study is subject to the following licenses/restrictions. The datasets generated in the current study are available from the corresponding author on reasonable request. Requests to access these datasets should be directed at: MP, m.peppelenbosch@erasmusmc.nl.

## Author Contributions

JS, ZLi, ZM, and MP conceived, designed, and supervised the study. YS performed the data collection. YS and JS developed the analysis pipeline and carried out the data process and visualization. FL, XT, ZLiu, GD, and JB contributed to the data analysis or interpretation. JS drafted the final manuscript. MP contributed to critical revision of the manuscript. All authors have read and approved the manuscript.

## Funding

This study was supported by the Changjiang Scholars and Innovative Research Team in University (IRT_17R88) and the Fundamental Research Funds for the Central Universities (31920180122).

## Conflict of Interest

The authors declare that the research was conducted in the absence of any commercial or financial relationships that could be construed as a potential conflict of interest.

## Publisher's Note

All claims expressed in this article are solely those of the authors and do not necessarily represent those of their affiliated organizations, or those of the publisher, the editors and the reviewers. Any product that may be evaluated in this article, or claim that may be made by its manufacturer, is not guaranteed or endorsed by the publisher.
