# Supplementary material for: Yak Gut Microbiota: A Systematic Review and Meta-Analysis
Source: Front Vet Sci. 2022 Jun 28;9:889594. doi: 10.3389/fvets.2022.889594 (PMC9274166; doi:10.3389/fvets.2022.889594)
Supplement: Supplementary file 2 [file Data_Sheet_1.docx]

**Supplementary figures**

**Figure S1.**


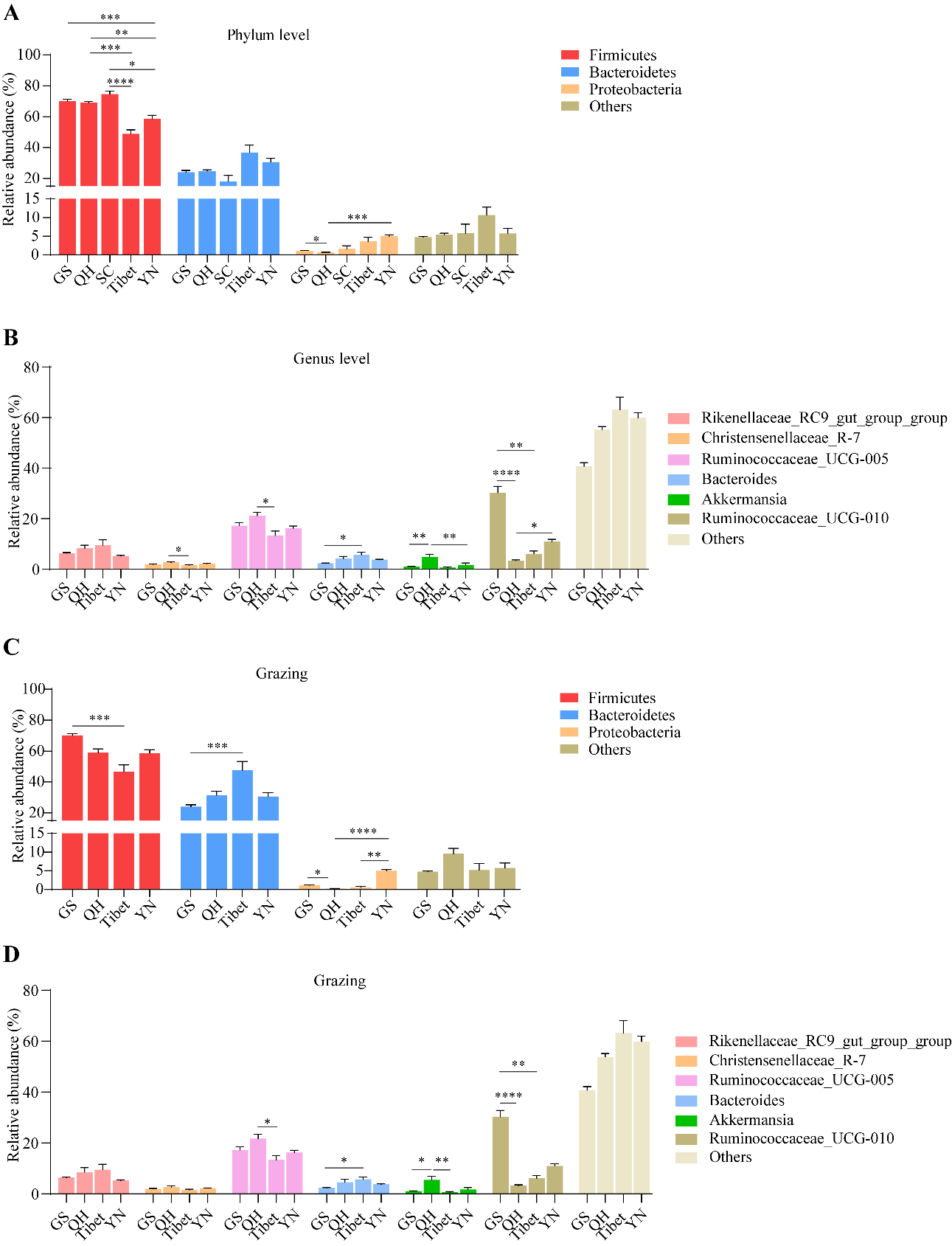


**Figure S1. Regional influence on yak fecal microbiota.** The relative abundance at phylum **(A)** or genus level **(B)** was compared between different provinces, irrespective of the method of yak husbandry. The relative abundance at phylum **(C)** or genus level **(D)** in yak maintained under grazing condition was compared between different provinces. The data was expressed as mean ± S.E.M. The Kruskal-Wallis test and post hoc Dunn’s multiple comparison test was used for data analysis. *p <0.05, **p<0.01, ***p<0.001, ****p <0.0001. GS, Gansu province; QH, Qinghai province; SC, Sichuan province; YP, Yunnan province.

**Figure S2.**


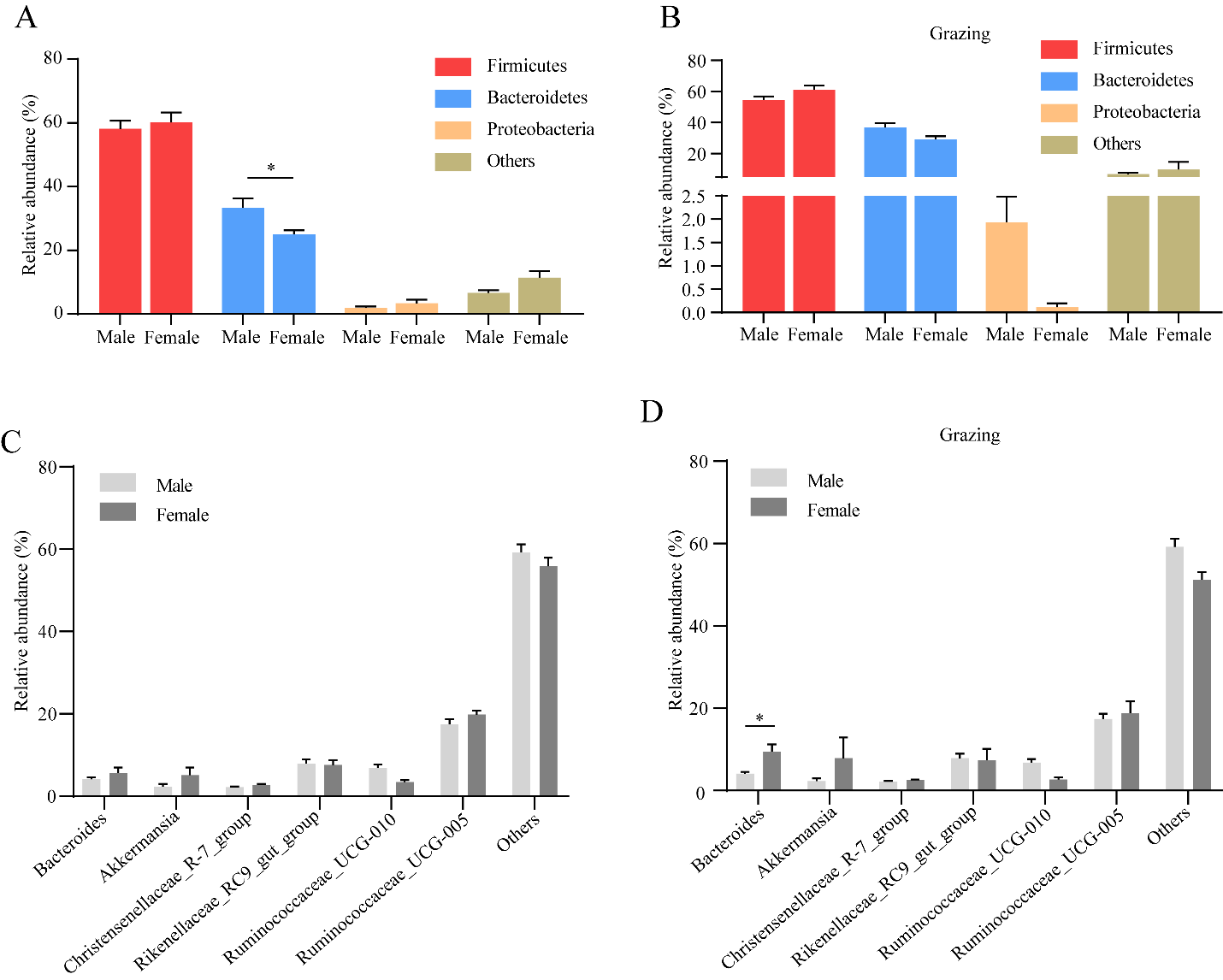


**Figure S2. Influence of sex on yak fecal microbiota. A**. The relative abundance at phylum level was compared between male and female yaks, irrespective of the method of yak husbandry. **B**. The difference for each phylum between male and female yaks maintained under grazing conditions. **C**. The relative abundance at genus level was compared between male and female yaks, irrespective of the method of yak husbandry. **D**. The difference of each genus between grazing male and female yaks. The data was expressed as mean ± S.E.M. The Mann-Whitney test was applied for statistical comparison. *p <0.05.

Figure S3.


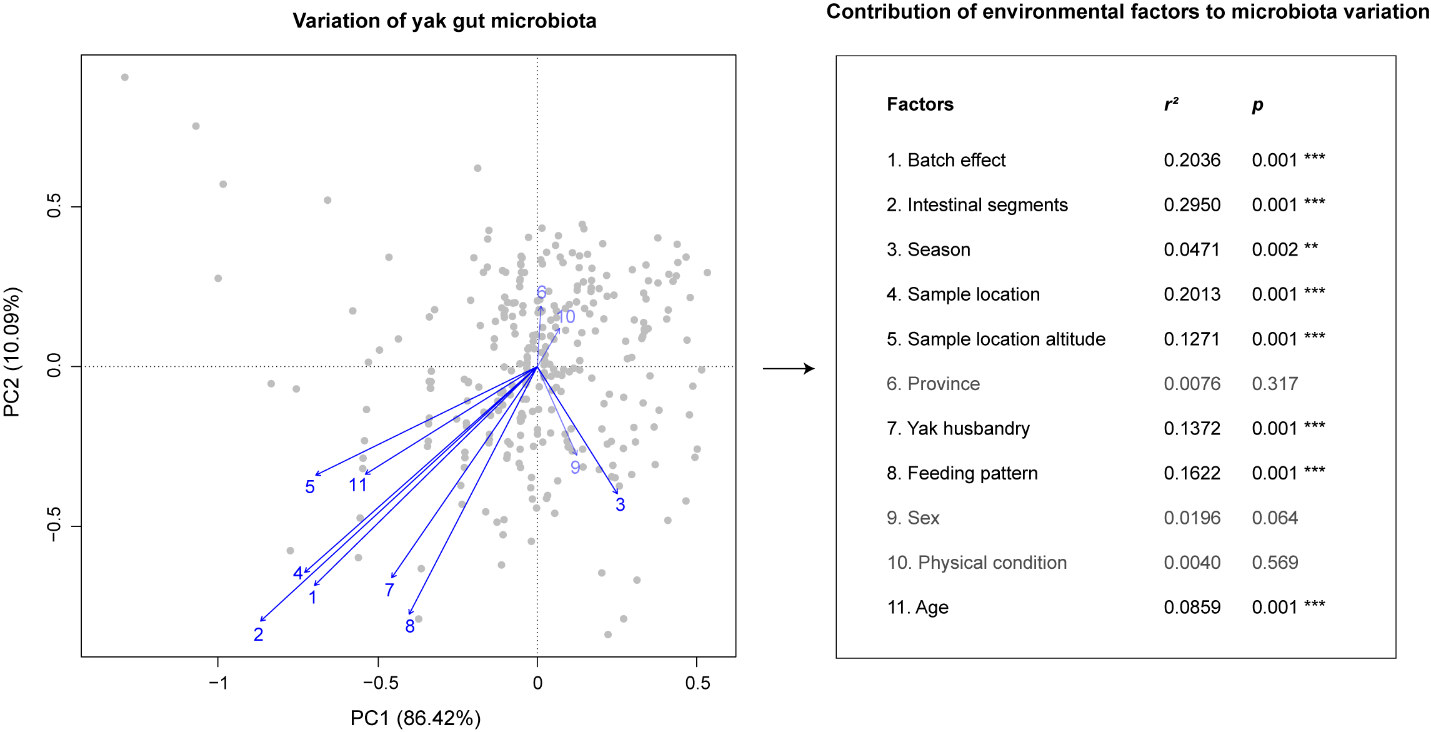


**Figure S3. The contribution of environmental factors to the variation of yak microbiota.** Variation in yak gut microbiota was displayed in a Bray-Curtis distance-based principal coordinate analysis (PCA) in the left panel. Each gray dot in PCA graph represents an individual sample. The envifit function in the R package vegan was used to determine the best linear fit for each environmental factors against the PCA ordination scores, and to test the significance of the fit using a permutation approach (perm=999).

**Supplementary Tables**

**Table S2.** Article searching strategy

| **Database searched** | **via** | **Years of coverage** | **References** | | **After de-duplication** | |
| --- | --- | --- | --- | --- | --- | --- |
| Embase | Embase.com | 1971 - Present | 69 | | 69 | |
| Medline ALL | Ovid | 1946 - Present | 57 | | 8 | |
| Web of Science Core Collection | Web of Knowledge | 1975 - Present | 97 | | 43 | |
| Cochrane Central Register of Controlled Trials | Wiley | 1992 - Present | 2 | | 1 | |
| Other sources: Google Scholar | | | | 10 | | 2 |
| **Total** | | | | **235** | | **123** |

**Table S2.** Descriptive summary of included studies on yak gut microbiome based on high throughput sequencing

| **Lead Author,**  **Year of publication** | **Area** | **Sample resource,**  **sample size** | **Main findings** | |
| --- | --- | --- | --- | --- |
| **Rumen (n=481)** |  |  |  | |
| ([Guo et al., 2015](#_ENREF_11)) | Sichuan | Rumen fluid, n=6 | Bacteroidetes and Firmicutes were the predominant phyla accounting for 39.68% and 45.90%, respectively. |  |
| ([Xue et al., 2016](#_ENREF_26)) | Sichuan | Rumen fluid, n=3 | The Bacteroidetes, 59.1% was the most abundant phylum, followed by Firmicutes and Proteobacteria. *Prevotella* was the predominant genus. |  |
| ([Xue et al., 2017](#_ENREF_27)) | Qinghai | Rumen fluid, n=8 | There was an obvious correlation of the community composition at the phylum and genus levels with the host or the feeding pattern. |  |
| ([Zhou et al., 2017](#_ENREF_33)) | Tibet | Rumen fluid, n=14 | The predominant bacterial phyla across feeding regimes were Bacteroidetes and Firmicutes. |  |
| ([Guo et al., 2018](#_ENREF_10)) | Gansu | Rumen fluid, n=6 | Rumen bacterial community in the 3 ruminant species differs. |  |
| ([Yan et al., 2018](#_ENREF_28)) | Gansu | Rumen fluid, n=6 | Increasing Slow-release urea supply significantly decreased abundance of the phylum Chlorobi and genus *Rikenellaceae RC9*. |  |
| ([Hu et al., 2019](#_ENREF_15)) | Qinghai | Rumen content, n=4 | Cysteamine hydrochloride and active dry yeast addition in basal rations increased the rumen beneficial bacterial of growth-retarded yaks. |  |
| ([Liu et al., 2019](#_ENREF_16)) | Qinghai-Tibetan Plateau | Rumen fluid, n=58 | Bacteroidetes and Firmicutes were the predominant bacterial phyla in the yak rumen. |  |
| ([Ma et al., 2019](#_ENREF_19)) | Qinghai | Rumen fluid, n=30 | Significant differences were found in the composition of yak ruminal microorganisms during different forage growth stages. |  |
| ([Xin et al., 2019](#_ENREF_25)) | Tibet | Rumen content, n=9 | The phyla Bacteroidetes and Firmicutes were the dominated bacteria regardless of breeds and regions. |  |
| ([Zou et al., 2019](#_ENREF_34)) | Qinghai-Tibetan Plateau | Rumen fluid, n =24 | The rumen microorganisms and their metabolism pathways changed with feed supply. |  |
| ([Ahmad et al., 2020](#_ENREF_1)) | Gansu | Rumen fluid, n=15 | High Firmicutes and Bacteroidetes were recorded with increased dietary energy levels. |  |
| ([Fan et al., 2020a](#_ENREF_3)) | Gansu | Rumen fluid, n=36 | Microbial diversity is different in rumen at different phenological stages. |  |

**Table S2. (**Continued)

| **Lead Author,**  **Year of publication** | **Area** | **Sample resource,**  **sample size** | **Main findings** |
| --- | --- | --- | --- |
| ([Fan et al., 2020b](#_ENREF_4)) | Gansu | Rumen fluid, n=24 | Rumen microbiota may influence yak milk protein yield. |
| ([Fan et al., 2020c](#_ENREF_5)) | Gansu, Tibet | Rumen fluid, n=36 | The enhanced ability of yaks in utilizing herbage may be partly owing to a microbiota adaptation for more energy requirements in the harsh environment. |
| ([Guo et al., 2020](#_ENREF_12)) | Gansu | Rumen fluid, n=80 | Rumen microbiota varied through the growth of yaks from neonatal to adult. |
| ([Hu et al., 2020](#_ENREF_14)) | Qinghai | Rumen fluid, n=18 | Increasing dietary energy increased the ratio of Firmicutes to Bacteroidetes and stimulated *Succiniclasticum*, *Saccharofermentans*, *Ruminococcus*, and *Blautia*. |
| ([Ma et al., 2020](#_ENREF_18)) | Qinghai | Rumen content, n=6 | Bacterial community in the gastrointestinal tract of growth-retarded yaks was disrupted compared to their healthy counterparts. |
| ([Ren et al., 2020](#_ENREF_21)) | Gansu | Rumen fluid, n=5 | Bacterial communities were different between the liquid, solid, and epithelium fractions, and between dorsal and ventral epithelium fractions. |
| ([Wu et al., 2020](#_ENREF_23)) | Yunnan | Rumen fluid, n=6 | There were differences in the abundance and diversity of certain bacteria in the rumen of different breeds of cattle under the same dietary environment. |
| ([Yang et al., 2020](#_ENREF_29)) | Gansu | Rumen fluid, n=16 | Yaks grazing in a high shrub-coverage pasture may have improved dietary energy utilization and enhanced resistance to cold stress. |
| ([Ahmad et al., 2021](#_ENREF_2)) | Gansu | Rumen fluid, n=21 | Dietary supplementation influenced the rumen microbial composition of yak. |
| ([Wei et al., 2021](#_ENREF_22)) | Qinghai | Rumen fluid, n=16 | Astragalus root extract supplementation influenced the proportions of Firmicutes, Bacteroidetes, Actinobacteria and Proteobacteria in rumen microbiota. |
| ([Wu et al., 2021](#_ENREF_24)) | Yunnan | Rumen fluid, n=18 | Significant differences in Bacteroidetes, Firmicutes, Ascomycota, and Chytridiomycota in the rumen of yaks were evident in different regions. |
| ([Zhao et al., 2021](#_ENREF_32)) | Tibet | Rumen fluid, n=16 | Supplementation of rumen-protected methionine or lysine can affect rumen microbial populations. |

**Table S2. (**Continued)

| **Lead Author,**  **Year of publication** | **Area** | **Sample resource,**  **sample size** | **Main findings** |
| --- | --- | --- | --- |
| **Feces (n=417)** |  |  |  |
| ([Han et al., 2017](#_ENREF_13)) | Sichuan | Feces, n=4 | Significant differences at phylum and genus levels were observed between groups. |
| ([Fu et al., 2020](#_ENREF_6)) | Qinghai | Feces, n=57 | Host is a dominant factor in shaping the microbial communities in sympatric herbivorous mammals. |
| ([Gong et al., 2020](#_ENREF_8)) | Tibet | Feces, n=5 | The major microbial contributors to yak fecal microbiota were Bacteroidaceae, Ruminococcaceae, Rikenellaceae, Clostridiaceae, and Prevotellaceae. |
| ([Zhang et al., 2020b](#_ENREF_31)) | Qinghai | Feces, n=24 | Abrupt adjustment to diet is likely to influence the gut micro-ecology. |
| ([Fu et al., 2021](#_ENREF_7)) | Qinghai | Feces, n=9 | Wild and half-blood yaks harbor an increased Firmicutes and reduced *Akkermansia*. The gut microbial diversity decreased in domestic yaks. |
| ([Guo et al., 2021](#_ENREF_9)) | Gansu | Feces, n=300 | Turnover of seasonal diet and gut microbiota composition occurred consistently. |
| ([Liu et al., 2021](#_ENREF_17)) | Yunnan, Qinghai, Tibet | Feces, n=18 | Geographical conditions influence the diversity and composition of the intestinal microbiota of yaks. |
| **Others (n=84)** |  |  |  |
| ([Nie et al., 2017](#_ENREF_20)) | Sichuan | Colon content, n=15 | Firmicutes and Bacteroidetes varied greatly during yaks’ growth. |
| ([Xin et al., 2019](#_ENREF_25)) | Tibet | Reticulum content, n=9  Omasum content, n=9  Abomasum content, n=9 | The phyla Bacteroidetes and Firmicutes were the dominated bacteria regardless of breeds and regions. |
| ([Ma et al., 2020](#_ENREF_18)) | Qinghai | Duodenum content, n=6  Jejunum content, n=6  Ileum content, n=6  Cecum content, n=6  Colon content, n=6 | Bacterial community in the gastrointestinal tract of growth-retarded yaks was disrupted compared to their healthy counterparts. |
| ([Zhang et al., 2020a](#_ENREF_30)) | Qinghai | Duodenum content, n=3  Jejunum content, n=3  Ileum content, n=3  Cecum content, n=3 | Ruminococcaceae, Bacteroidaceae and Muribaculaceae were higher in cecum than in other segments of intestines. |

**Table S3**. Prevalence of yak gut bacteria in included samples

| Detected taxa (n) | Prevalence in more than 40% of samples (%) | Prevalence in less than 40% of samples (%) |  |  |  |
| --- | --- | --- | --- | --- | --- |
| Phylum | Firmicutes (82.5) | SR1 (7.8) | Acidobacteria (2.9) | Chloroflexi (2.9) | Lentisphaerae (21.4) |
| (24) | Bacteroidetes (83.5) | LGCGPB (1.9) | Tenericutes (35.9) | SHA-109 (2.9) | Actinobacteria (24.3) |
|  | Proteobacteria (69.9) | Archaea (1.0) | Spirochaetes (30.1) | Synergistetes (7.8) | Cyanobacteria (11.7) |
|  |  | TM7 (12.6) | Fibrobacteres (33) | Saccharibacteria (1.9) | Verrucomicrobia (16.5) |
|  |  | Chordata (2.9) | Low G + C Subdivision (1.0) | Epsilonbacteraeota (1.0) | Euryarchaeota (20.4) |
|  |  |  |  |  | Cytophaga-Flexibacter-Bacteroides (1.9) |
| Class |  | Bacilli (1.0) | WCHB1_41 (2.9) | Bacteroidia (7.8) | Methanobacteria (3.9) |
| (19) |  | Clostridia (7.8) | Mollicutes (6.8) | Spirochaetia (6.8) | Alphaproteobacteria (3.9) |
|  |  | LD1_PB3 (2.9) | Flavobacteria (1.0) | Lentisphaeria (6.8) | Betaproteobacteria (1.0) |
|  |  | Erysipelotrichia (3.9) | Epsilonproteobacteria (1.0) | Negativicutes (2.9) | Thermoplasmata (1.0) |
|  |  |  | Deltaproteobacteria (3.9) | Alphaproteobacteria (1.0) | Gammaproteobacteria (1.0) |
| Order |  | Victivallales (2.9) | Bacteroidales (4.9) | Lactobacillales (1.0) | Bradymonadales (2.9) |
| (13) |  | Spirochaetales (2.9) | Clostridiales (4.9) | Fusobacteriales (1.0) | Erysipelotrichales (2.9) |
|  |  | Enterobacteriales (1.0) | Selenomonadales (2.9) | Oligosphaerales (2.9) | Gastranaerophilales (2.9) |
|  |  |  |  |  | Anaeroplasmatales (2.9) |
| Family |  | BS11 (1.9) | Lachnospiraceae (21.4) | Rikenellaceae (2.9) | Fusobacteriaceae (1.0) |
| (24) |  | Family_XIII (2.9) | Ruminococcaceae (23.3) | Planococcaceae (1.0) | Peptostreptococcaceae (16.5) |
|  |  | Prevotellaceae (21.4) | Bacteroidaceae (17.5) | Veillonellaceae (2.9) | Bacteroidales_S24-7_group (2.9) |
|  |  | Flavobacteriaceae (15.5) | Paraprevotellaceae (1.0) | Christensenellaceae (2.9) | Bacteroidales_RF16_group (6.8) |
|  |  | Muribaculaceae (15.5) | Succinivibrionaceae (15.5) | Erysipelotrichaceae (1.0) | Unidentified_Flavobacteriales (11.7) |
|  |  | Moraxellaceae (15.5) | Unclassified Clostridiales (1.0) | Unclassified Bacteroidales (1.9) | Bacteroidales_BS11_gut_group (2.9) |
|  |  |  |  |  |  |
| Genus | Succiniclasticum (45.6) | Treponema (16.5) | Bifidobacterium (2.9) | Blautia (5.8) | Saccharofermentans (15.5) |
| (160) | Prevotella (40.8) | Turicibacter (4.9) | Olsenella (2.9) | Faecalibacterium (1.0) | vadinCA11 (13.6) |
|  | Fibrobacter (40.8) | Ruminococcus (15.5) | Senegalimassilia (2.9) | Treponema_2 (17.5) | Escherichia/Shigella (5.8) |
|  |  | Butyrivibrio_2 (19.4) | Desulfovibrio (9.7) | Ruminococcus_1 (21.4) | Clostridium_sensu_stricto (14.6) |
|  |  | Lachnoclostridium (4.9) | Prevotella_1 (22.3) | Papillibacter (19.4) | Methanobrevibacter (17.5) |
|  |  | Unclassified RFP12 (9.7) | unclassified BS11 (10.7) | Pseudobutyrivibrio (5.8) | Christensenellaceae_R-7_group (17.5) |
|  |  | Unclassified F16 (1.9) | Clostridium XI (4.9) | Selenomonas_1 (6.8) | Christensenellaceae R7 (11.7) |
|  |  | Howardella (3.9) | Acetivibrio (1.0) | Campylobacter (3.9) | Ruminococcaceae_NK4A214_group (23.3) |
|  |  | Aeriscardovia (2.9) | Bacteroides (20.4) | Clostridium_XIVa (1.9) | Ruminococcaceae_UCG-010 (15.5) |
|  |  | Oscillibacter (2.9) | Lactobacillus (1.0) | Clostridium_XIVb (2.9) | Ruminococcaceae_UCG-005 (21.4) |
|  |  | Clostridium_IV (1.9) | Butyrivibrio (16.5) | CF231 (12.6) | Eubacterium_coprostanoligenes_group (10.7) |
|  |  | Romboutsia (17.5) | Anaeroplasma (5.8) | YRC22 (12.6) | Ruminococcaceae_UCG-014 (11.7) |
|  |  | Acetobacter (5.8) | Mycoplasma (2.9) | SHD-231 (1.9) | Rikenellaceae_RC9_gut_group (33.0) |
|  |  | Solibacillus (3.9) | Atopobium (4.9) | Dehalobacterium (1.9) | Prevotellaceae_UCG-003 (33.0) |
|  |  | BF311 (17.5) | Anaerofustis (3.9) | Moryella (1.9) | Prevotellaceae_UCG-001 (33.0) |
|  |  | Bulleidia (1.9) | Burkholderia (5.8) | Shuttleworthia (1.9) | Lachnospiraceae_ND3007_group (5.8) |
|  |  |  |  |  |  |
| **Table S3.** (Continued) | |  |  |  |  |
|  |  | TG5 (1.0) | Serratia (1.0) | Oscillospira (5.8) | Unclassified Victivallaceae (1.9) |
|  |  | Flavobacterium (15.5) | Raistonia (1.0) | Anaerovibrio (1.9) | Unclassified Christensenellaceae (1.9) |
|  |  | Sphaerochaeta (1.9) | Victivallis (1.0) | Clostridium III (1.9) | Lachnospiraceae AC2044 (5.8) |
|  |  | Psychrobacter (15.5) | Sharpea (1.0) | horsej‐a03 (3.9) | Erysipelotrichaceae UCG 004 (3.9) |
|  |  | Pseudomonas (14.6) | Limnobacter (1.0) | Quinella (3.9) | Blvii28 wastewater‐sludge group (3.9) |
|  |  | Succinivibrio (15.5) | Kandieria (1.0) | Roseburia (3.9) | unclassified Ruminococcaceae (11.7) |
|  |  | Clostridium (2.9) | Thermus (1.0) | Comamonas (3.9) | unclassified Lachnospiraceae (10.7) |
|  |  | Thauera (5.8) | Dialister (1.0) | Solobacterium (3.9) | unclassified “Fusobacteriaceae” (1.0) |
|  |  | Arcobacter (2.9) | Alistipes (1.0) | Family XIII AD3011 (3.9) | unclassified “Prevotellaceae” (10.7) |
|  |  | Aquabacterium (1.0) | Anaerovorax (3.9) | GCA−900066575 (3.9) | Lachnospiraceae_XPB1014_group (10.7) |
|  |  | Stomatobaculum (1.0) | Mailhella (3.9) | Sutterella (3.9) | Unclassified Pirellulaceae (1.9) |
|  |  | Faecailbacterium (1.0) | Pelobacter (3.9) | Syntrophococcus (4.9) | Lachnospiraceae UCG 008 (15.5) |
|  |  | Parabacteroides (1.0) | Enterorhabdus (3.9) | Methylobacterium (1.0) | Ruminococcaceae UCG 004 (3.9) |
|  |  | Vulcanlibacterum (1.0) | Fretibacterium (3.9) | Acetitomaculum (3.9) | Ruminococcaceae UCG 009 (3.9) |
|  |  | Alloprevotella (26.2) | Tyzzerella 3 (3.9) | Pseudobacteroides (3.9) | Lachnospiracea_incertae_sedis (1.0) |
|  |  | Mogibacterium (9.7) | RFN20 (13.6) | p−1088‐a5 gut group (3.9) | Succinivibrionaceae_UCG_002 (11.7) |
|  |  | Gluconacetobacter (1.0) | Anaerostipes (5.8) | Lachnospiraceae NK4A136 (3.9) | Lachnospiraceae FCS020 (6.8) |
|  |  | p-75-a5 (5.8) | Selenomonas (5.8) | Lachnospiraceae UCG 006 (3.9) | Lachnospiraceae NK3A20 (3.9) |
|  |  | L7A_E11 (5.8) | Ruminobacter (9.7) | Chryseobacterium (13.6) | Prevotellaceae NK3B31 (8.7) |
|  |  | Pyramidobacter (1.9) | Asteroleplasma (1.0) | Prevotellaceae UCG-004 (5.8) | Eubacterium coprostanoligenes (5.8) |
|  |  | probable genus 10 (3.9) | Methanosphaera (2.9) | Veillonellaceae UCG 001 (3.9) | Unclassified Veillonellaceae (9.7) |
|  |  | Ruminiclostridium 6 (3.9) | Acinetobacter (16.5) | Lachnospiraceae AC2044 (5.8) | Unclassified Mogibacteriaceae (1.9) |
|  |  | Sphingomonas (13.6) | Unclassified S24-7 (9.7) | Methanimicrococcus (1.0) | Lachnospiraceae UCG 010 (3.9) |
|  |  |  |  |  | Unclassified Dethiosulfovibrionaceae (1.0) |
| Species |  | Prevotella spp. (1.0) | Prevotella ruminicola (1.0) | Ruminococcus gauvreauii (3.9) | Methanobrevibacter gottschalki (1.0) |
| (16) |  | Prevotella brevis (3.9) | Eubacterium nodatum (3.9) | Pseudobutyrivibrio ruminis (1.0) | Butyrivibrio fibrisolvens C219a (1.0) |
|  |  | Ruminococcus albus (3.9) | Succiniclasticum ruminis (1.0) | Ruminococcus flavefaciens (4.9) | Clostridiales bacterium DJF_VP48 (1.0) |
|  |  | Prevotella ruminicola (1.0) | Fibrobacter succinogenes (3.9) | Selenomonas ruminantium (4.9) | Schwartzia succinivorans DSM 10502T (1.0) |
|  |  |  |  |  |  |
|  |  |  |  |  |  |

**Supplementary references**

Ahmad, A.A., Yang, C., Zhang, J., Kalwar, Q., Liang, Z., Li, C., Du, M., Yan, P., Long, R., Han, J., Ding, X., 2020. Effects of Dietary Energy Levels on Rumen Fermentation, Microbial Diversity, and Feed Efficiency of Yaks (Bos grunniens). Front Microbiol 11.

Ahmad, A.A., Zhang, J.B., Liang, Z., Yang, C., Kalwar, Q., Shah, T., Du, M., Muhammad, I., Zheng, J., Yan, P., Ding, X.Z., Long, R., 2021. Dynamics of rumen bacterial composition of yak (Bos grunniens) in response to dietary supplements during the cold season. PeerJ 9.

Fan, Q., Wanapat, M., Hou, F., 2020a. Chemical Composition of Milk and Rumen Microbiome Diversity of Yak, Impacting by Herbage Grown at Different Phenological Periods on the Qinghai-Tibet Plateau. Animals 10.

Fan, Q., Wanapat, M., Hou, F., 2020b. Rumen bacteria influence milk protein yield of yak grazing on the Qinghai-Tibet Plateau.

Fan, Q., Wanapat, M., Yan, T., Hou, F., 2020c. Altitude influences microbial diversity and herbage fermentation in the rumen of yaks. BMC Microbiol 20.

Fu, H., Zhang, L., Fan, C., Liu, C., Li, W., Cheng, Q., Zhao, X., Jia, S., Zhang, Y., 2020. Environment and host species identity shape gut microbiota diversity in sympatric herbivorous mammals. Microb Biotechnol.

Fu, H., Zhang, L., Fan, C., Liu, C., Li, W., Li, J., Zhao, X., Jia, S., Zhang, Y., 2021. Domestication Shapes the Community Structure and Functional Metagenomic Content of the Yak Fecal Microbiota. Front Microbiol 12.

Gong, G., Zhou, S., Luo, R., Gesang, Z., Suolang, S., 2020. Metagenomic insights into the diversity of carbohydrate-degrading enzymes in the yak fecal microbial community. BMC Microbiol 20.

Guo, N., Wu, Q., Shi, F., Niu, J., Zhang, T., Degen, A.A., Fang, Q., Ding, L., Shang, Z., Zhang, Z., Long, R., 2021. Seasonal dynamics of diet–gut microbiota interaction in adaptation of yaks to life at high altitude. npj Biofilms and Microbiomes 7.

Guo, W., Bi, S., Kang, J., Zhang, Y., Long, R., Huang, X., Shan, M.N., Anderson, R.C., 2018. Bacterial communities related to 3-nitro-1-propionic acid degradation in the rumen of grazing ruminants in the Qinghai-Tibetan Plateau. Anaerobe 54, 42-54.

Guo, W., Li, Y., Wang, L., Wang, J., Xu, Q., Yan, T., Xue, B., 2015. Evaluation of composition and individual variability of rumen microbiota in yaks by 16S rRNA high-throughput sequencing technology. Anaerobe 34, 74-79.

Guo, W., Zhou, M., Ma, T., Bi, S., Wang, W., Zhang, Y., Huang, X., Guan, L.L., Long, R., 2020. Survey of rumen microbiota of domestic grazing yak during different growth stages revealed novel maturation patterns of four key microbial groups and their dynamic interactions. Anim. microbiome 2, 23.

Han, Z., Li, K., Shahzad, M., Zhang, H., Luo, H., Qiu, G., Lan, Y., Wang, X., Mehmood, K., Li, J., 2017. Analysis of the intestinal microbial community in healthy and diarrheal perinatal yaks by high-throughput sequencing. Microb Pathog 111, 60-70.

Hu, R., Zou, H., Wang, H., Wang, Z., Wang, X., Ma, J., Shah, A.M., Peng, Q., Xue, B., Wang, L., Zhao, S., Kong, X., 2020. Dietary energy levels affect rumen bacterial populations that influence the intramuscular fat fatty acids of fattening yaks (Bos grunniens). Animals 10, 1-16.

Hu, R., Zou, H., Wang, Z., Cao, B., Peng, Q., Jing, X., Wang, Y., Shao, Y., Pei, Z., Zhang, X., Xue, B., Wang, L., Zhao, S., Zhou, Y., Kong, X., 2019. Nutritional interventions improved rumen functions and promoted compensatory growth of growth-retarded yaks as revealed by integrated transcripts and microbiome analyses. Front Microbiol 10.

Liu, C., Wu, H., Liu, S., Chai, S., Meng, Q., Zhou, Z., 2019. Dynamic alterations in yak rumen bacteria community and metabolome characteristics in response to feed type. Front Microbiol 10.

Liu, W., Wang, Q., Song, J., Xin, J., Zhang, S., Lei, Y., Yang, Y., Xie, P., Suo, H., 2021. Comparison of Gut Microbiota of Yaks From Different Geographical Regions. Front Microbiol 12.

Ma, J., Zhu, Y., Wang, Z., Yu, X., Hu, R., Wang, X., Cao, G., Zou, H., Shah, A.M., Peng, Q., Xue, B., Wang, L., Zhao, S., Kong, X., 2020. Comparing the Bacterial Community in the Gastrointestinal Tracts Between Growth-Retarded and Normal Yaks on the Qinghai–Tibetan Plateau. Front Microbiol 11.

Ma, L., Xu, S., Liu, H., Xu, T., Hu, L., Zhao, N., Han, X., Zhang, X., 2019. Yak rumen microbial diversity at different forage growth stages of an alpine meadow on the Qinghai-Tibet Plateau. PeerJ 2019.

Nie, Y., Zhou, Z., Guan, J., Xia, B., Luo, X., Yang, Y., Fu, Y., Sun, Q., 2017. Dynamic changes of yak (Bos grunniens) gut microbiota during growth revealed by polymerase chain reaction-denaturing gradient gel electrophoresis and metagenomics. Asian-australas j anim sci 30, 957-966.

Ren, Q., Si, H., Yan, X., Liu, C., Ding, L., Long, R., Li, Z., Qiu, Q., 2020. Bacterial communities in the solid, liquid, dorsal, and ventral epithelium fractions of yak (Bos grunniens) rumen. MicrobiologyOpen 9.

Wei, H., Ding, L., Wang, X., Yan, Q., Jiang, C., Hu, C., Wang, G., Zhou, Y., Henkin, Z., Degen, A.A., 2021. Astragalus root extract improved average daily gain, immunity, antioxidant status and ruminal microbiota of early weaned yak calves. J Sci Food Agric 101, 82-90.

Wu, D., Vinitchaikul, P., Deng, M., Zhang, G., Sun, L., Gou, X., Mao, H., Yang, S., 2020. Host and altitude factors affect rumen bacteria in cattle. Braz J Microbiol 51, 1573-1583.

Wu, D., Vinitchaikul, P., Deng, M., Zhang, G., Sun, L., Wang, H., Gou, X., Mao, H., Yang, S., 2021. Exploration of the effects of altitude change on bacteria and fungi in the rumen of yak (Bos grunniens). Arch Microbiol 203, 835-846.

Xin, J., Chai, Z., Zhang, C., Zhang, Q., Zhu, Y., Cao, H., Zhong, J., Ji, Q., 2019. Comparing the microbial community in four stomach of dairy cattle, yellow cattle and three yak herds in qinghai-tibetan plateau. Front Microbiol 10.

Xue, D., Chen, H., Chen, F., He, Y.X., Zhao, C., Zhu, D., Zeng, L.L., Li, W., 2016. Analysis of the rumen bacteria and methanogenic archaea of yak (Bos grunniens) steers grazing on the Qinghai-Tibetan Plateau. Livest. Sci. 188, 61-71.

Xue, D., Chen, H., Zhao, X., Xu, S., Hu, L., Xu, T., Jiang, L., Zhan, W., 2017. Rumen prokaryotic communities of ruminants under different feeding paradigms on the Qinghai-Tibetan Plateau. Syst Appl Microbiol 40, 227-236.

Yan, X.T., Yan, B.Y., Ren, Q.M., Dou, J.J., Wang, W.W., Zhang, J.J., Zhou, J.W., Long, R.J., Ding, L.M., Han, J., Li, Z.P., Qiu, Q., 2018. Effect of slow-release urea on the composition of ruminal bacteria and fungi communities in yak. Anim. Feed Sci. Technol. 244, 18-27.

Yang, C., Tsedan, G., Liu, Y., Hou, F., 2020. Shrub coverage alters the rumen bacterial community of yaks (Bos grunniens) grazing in alpine meadows. J. anim. sci. technol. 62, 504-520.

Zhang, L., Jiang, X., Li, A., Waqas, M., Gao, X., Li, K., Xie, G., Zhang, J., Mehmood, K., Zhao, S., Wangdui, B., Li, J., 2020a. Characterization of the microbial community structure in intestinal segments of yak (Bos grunniens). Anaerobe 61.

Zhang, X.L., Xu, T.W., Wang, X.G., Geng, Y.Y., Liu, H.J., Hu, L.Y., Zhao, N., Kang, S.P., Zhang, W.M., Xu, S.X., 2020b. The effect of transitioning between feeding methods on the gut microbiota dynamics of yaks on the qinghai–tibet plateau. Animals 10, 1-14.

Zhao, Z.W., Ma, Z.Y., Wang, H.C., Zhang, C.F., 2021. Effects of rumen-protected methionine and lysine supplementation on milk yields and components, rumen fermentation, and the rumen microbiome in lactating yaks (Bos grunniens). Animal Feed Science and Technology 277.

Zhou, Z., Fang, L., Meng, Q., Li, S., Chai, S., Liu, S., Schonewille, J.T., 2017. Assessment of ruminal bacterial and archaeal community structure in Yak (Bos grunniens). Front Microbiol 8.

Zou, H., Hu, R., Wang, Z., Shah, A.M., Zeng, S., Peng, Q., Xue, B., Wang, L., Zhang, X., Wang, X., Shi, J., Li, F., Zeng, L., 2019. Effects of nutritional deprivation and re-alimentation on the feed efficiency, blood biochemistry, and rumen microflora in yaks (Bos grunniens). Animals 9.
